# Molecular Evolutionary and Expression Pattern Analysis of *AKR* Genes Shed New Light on *GalUR* Functional Characteristics in *Brassica rapa*

**DOI:** 10.3390/ijms21175987

**Published:** 2020-08-20

**Authors:** Weike Duan, Zhinan Huang, Ying Li, Xiaoming Song, Xiaochuan Sun, Cong Jin, Yunpeng Wang, Jizhong Wang

**Affiliations:** 1 College of Life Sciences and Food Engineering, Huaiyin Institute of Technology, Huai’an 223003, China; weikeduan@126.com (W.D.); sunxch@hyit.edu.cn (X.S.); jincong@hyit.edu.cn (C.J.); ypwang@hyit.edu.cn (Y.W.); hgxjz@hyit.edu.cn (J.W.); 2 State Key Laboratory of Crop Genetics and Germplasm Enhancement, Key Laboratory of Biology and Germplasm Enhancement of Horticultural Crops in East China, College of Horticulture of Nanjing Agricultural University, Nanjing 210095, China; 3 School of Life Science and Center for Genomics and Computational Biology, North China University of Science and Technology, Tangshan 063210, China; songxm@ncst.edu.cn

**Keywords:** AKR, GalUR, evolutionary history, expression pattern, selective pressure, *Brassica rapa*

## Abstract

The aldo-keto reductase (AKR) superfamily plays a major role in oxidation-reduction in plants. _D_-galacturonic acid reductase (GalUR), an ascorbic acid (AsA) biosynthetic enzyme, belongs to this superfamily. However, the phylogenetic relationship and evolutionary history of the AKR gene family in plants has not yet been clarified. In this study, a total of 1268 *AKR* genes identified in 36 plant species were used to determine this phylogenetic relationship. The retention, structural characteristics, and expression patterns of *AKR* homologous genes in *Brassica rapa* and *Arabidopsis thaliana* were analyzed to further explore their evolutionary history. We found that the AKRs originated in algae and could be divided into A and B groups according to the bootstrap value; GalURs belonged to group A. Group A *AKR* genes expanded significantly before the origin of angiosperms. Two groups of *AKR* genes demonstrated functional divergence due to environmental adaptability, while group A genes were more conservative than those in group B. All 12 candidate GalUR genes were cloned, and their expression patterns under stress were analyzed, in Pak-choi. These genes showed an obvious expression divergence under multiple stresses, and *BrcAKR22* exhibited a positive correlation between its expression trend and AsA content. Our findings provide new insights into the evolution of the AKR superfamily and help build a foundation for further investigations of *GalUR*’s functional characteristics.

## 1. Introduction

In nature, plants unavoidably encounter environmental insults, including various abiotic and biotic stresses. However, under these situations, plants have also developed a myriad of defense strategies. l-ascorbic acid (AsA), a multifunctional molecule, is an essential metabolite for plants, with roles as an antioxidant, enzyme cofactor, redox signaling modulator, etc. [1,2]. To date, four biosynthetic routes for AsA have been proposed, including the _L_-galactose pathway, the myo-Inositol/glucuronate pathway, the _L_-gulose pathway, and the galacturonate pathway [3,4,5]. The identified key enzymes in these biosynthetic pathways greatly contribute to the total AsA pool in specific plants and significantly improve their resistance [6,7].

The aldo-keto reductases (*AKR*) gene was first identified to encode _D_-galacturonic acid reductase (GalUR) in a strawberry over 17 years ago and has been proved to play an important role in the AsA biosynthesis [5,6]. Meanwhile, transgenic tomato plants expressing strawberry *GalUR* also had enhanced tolerance to abiotic stresses [8]. In the present study, most *GalUR* genes were discovered based on their sequence alignment. All 18 *GalUR* paralogs were identified in the citrus genome [9]; subsequently, 17 *GalUR* paralogs were identified in the kiwifruit genome [10]. Notably, the *GalUR* homologs showed diverse expression patterns across different developmental stages in these two species, but only *Citrus GalUR12* was significantly upregulated in fruit and could be confirmed as a contributor to AsA accumulation in orange fruit [9,11].

All GalUR protein sequences that were analyzed shared significant homology with cytosolic NAD(P)H-dependent aldo-keto reductase (AKR) with the specific domain Aldo-ket_red (PF00248) [12]. AKR comprises a diverse family of enzymes that catalyze the reduction of carbonyl compounds to the corresponding alcohols (or engage in reverse oxidation) and are widely present in fungi, animals, and plants [13]. The functional importance of some *AKR* genes has been well characterized in plants, and AKRs can be broadly categorized into four important functional groups that reflect their major functional roles in plant metabolism and development, including reactive aldehyde detoxification, secondary metabolism, the biosynthesis of osmolytes, and membrane transport [13]. In addition, plant *AKR* genes, such as *AKR4C9,* play an important role in reductive detoxification under abiotic or biotic stress conditions [14]. The functional differentiation of multigene family members might provide more flexibility to adapt to different environments. A previous analysis of *Arabidopsis* suggested that its genome contains at least 21 *AKR* homologs [14], though only seven such members can be identified as *GalUR* genes [9]. However, previous studies mainly focused on the structural and functional aspects of plant *AKRs* and their multiple significant roles in diverse plant metabolic processes [13]. The molecular evolution and structural analyses of *AKRs*, however, have mainly focused on humans [15,16].

How many distinct groups of *AKR* genes are there in plants? What is their evolutionary relationship? Were *GalUR* genes a subgroup of *AKR* genes? According to previous reports, not all *GalUR* homologs contributed to the AsA content in plants, and it appears that the role of GalUR proteins in AsA biosynthesis might be unique [12]. Do the neofunctionalization or subfunctionalization models apply to these genes? To answer these questions, we performed the following analyses: (i) phylogenetic; (ii) nucleotide distance; (iii) sequence characteristics; (iv) selective pressure; (v) retention; and (vi) expression patterns. The results provide a basis for understanding the evolutionary history of *AKR* genes and will help further elucidate the functional characteristics of *GalUR*.

## 2. Results

### 2.1. Phylogenetic Relationship of AKR Genes

**Figure 1:**
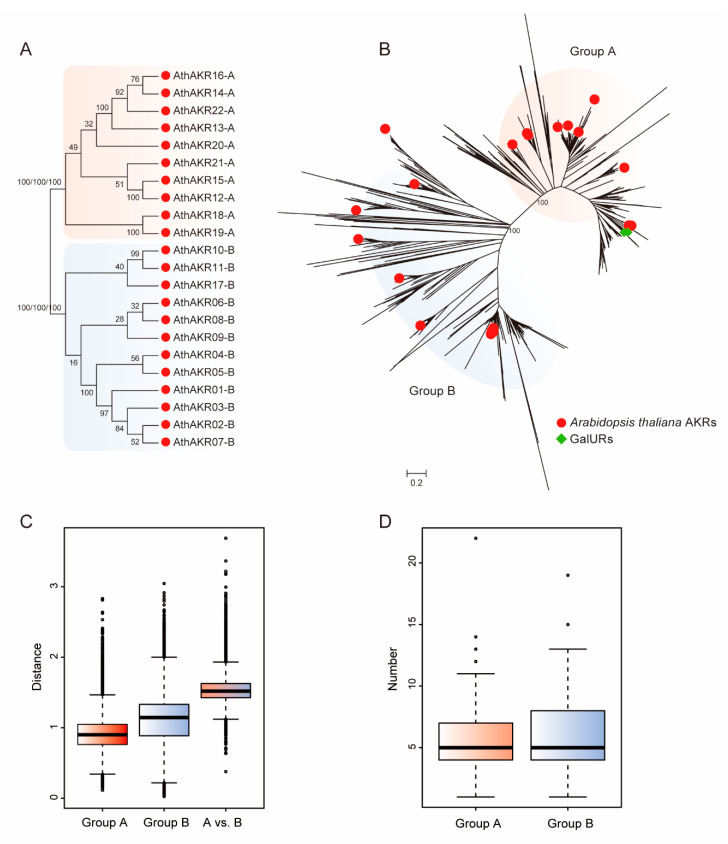
Phylogenetic relationship of the aldo-keto reductases (AKR) gene family and the characteristics of group A and B genes. (**A**) Phylogenetic tree of AKRs in *A. thaliana*. The tree was constructed using the maximum-likelihood (ML) method and examined by the maximum-parsimony (MP) and neighbor-joining (NJ) methods. The bootstrap values were calculated with 1000 replications. (**B**) A phylogenetic tree of all 1268 AKRs in the selected 36 plant species with the known GalURs. Red circles represent *A. thaliana* AKRs, and green boxes represents GalURs. (**C**) Nucleotide distance of group A and B *AKR* genes. (**D**) The boxplots of exon numbers in group A and B *AKR* genes.

To clarify the phylogenetic relationships of the AKRs, we selected 36 representative plants, including 6 algae, 1 bryophyte, 1 lycophyte, and 28 angiosperm plants (Supplementary Table S1). Algae are the ancestors of land plants, and bryophytes are the closest extant relatives of early land plants. Lycophytes are early vascular plants with a dominant sporophyte generation, and angiosperm plants possess more complex organ systems and structures. A total of 1268 AKRs were identified in the 36 selected plant species after preliminary screening by the Hidden Markov Model (HMM) software package and verified by the Pfam, SMART, and NCBI databases (Supplementary Table S2). In total, 22 *AKR* genes were identified in *A. thaliana*—one more than found in the previous report [14]. To determine the subgroups of the AKR gene family, the following analyses were performed. Firstly, the phylogenetic relationships among 22 *A. thaliana* AKR proteins were reconstructed with the maximum-likelihood (ML) neighbor-joining (NJ) and maximum-parsimony (MP) methods. The results showed that the AKRs clearly formed two distinct groups (group A and B) according to the bootstrap value (Figure 1A). Then, the phylogenetic relationships among all 1268 AKR proteins were reconstructed. These results also showed the AKRs divided into two distinct groups (Figure 1B). Lastly, the nucleotide distances among the two groups were calculated. The mean nucleotide distance of the group A and B *AKRs* was respectively smaller than that between the group A and B *AKRs* (Figure 1C). These observations demonstrate that the AKRs formed two distinct subgroups.

Furthermore, when building the phylogenetic trees for all AKRs, three known GalUR proteins (GenBank: AF039182, AY663110, and DQ843600) were analyzed together. Interestingly, the GalURs were clearly assigned to group A (Figure 1B).

Finally, we compared the exon number differences of the *AKR* genes between these two groups. The boxplot showed that their difference was not large (Figure 1D). The exons in the group B *AKR* genes had a wider range than those in group A. Meanwhile, the internal nucleotide difference in group B was larger than that in group A, preliminarily indicating that the genes in group A were relatively conservative (Figure 1C,D).

### 2.2. Evolutionary History and Structural Characteristics of the AKR Gene Family in Plants

**Figure 2:**
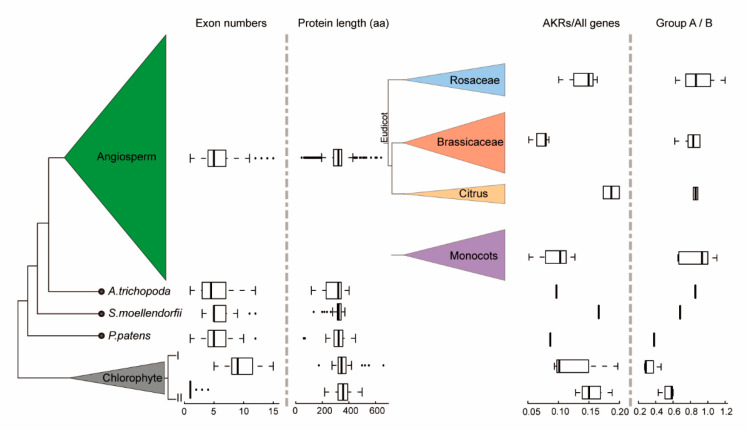
Distribution of the structural characteristics and numerical statistics of the aldo-keto reductases (AKR) gene family in plant species. From left to right: (i) the phylogenetic relationship of higher plants and algae; (ii) boxplots of the exon numbers of *AKR* genes; (iii) boxplots of the lengths of AKR proteins; (iv) the selected monocots and dicots in angiosperms; (v) boxplots of the percentage of *AKR* genes occupying all genes; (vi) boxplots of the ratio between group A and B *AKR* genes.

To further clarify the evolutionary history of the AKR gene family in plants, we first focus on the characteristics (exons and introns) of the gene sequences among the different types of plant groups, especially the algae and land plants. In algae, the distribution of the number of exons presents a visible divergence and can clearly be divided into two categories (Figure 2). In the early algae, the gene structure was relatively simple; most *AKRs* contained only one exon, such as *Ostreococcus lucimarinus* or *Micromonas pusilla*, while the number of exons increased significantly (the average number reached was nine) in *Coccomyxa subellipsoidea*, *Volvox carteri,* and *Chlamydomonas reinhardtii* (Supplementary Figure S1). From moss to higher plants, the number of exons was slightly reduced and stabilized at five (Figure 2). The smallest number of exons in higher plants with a large span was 1, and the largest was 22 (Supplementary Table S2). In all the selected plants, the protein sequence lengths of AKR were relatively stable and were mainly focused around 300–400 aa, while the lengths of the AKR proteins also had a large span (Figure 2).

Next, we compared the number of *AKR* genes in the plants, and the difference was obvious (Supplementary Figure S1). In algae, the number of *AKR* genes was relatively small, and in land plants, the number started to increase, especially in higher plants, where the number reached 101 (*Panicum virgatum*) (Supplementary Figure S1). Due to diverse evolutionary events, the total number of genes in each plant was different. Thus, understanding the proportion of *AKR* genes could better reveal the differences of *AKR* genes among the plants. However, there were significant differences in the ratio of *AKR* genes to all genes of each plant among the different types of plants (Figure 2 and Figure S1). In the selected citrus plants, the mean proportion of *AKRs* reached its maximum, and in the selected rosacea plants, the proportion of *AKR* genes was also relatively large (Figure 2).

Thirdly, to further explore the divergences between group A and group B *AKRs*, we compared the ratio between their gene numbers. Interestingly, the proportion of group A compared to group B *AKRs* showed an increasing trend from the algae to higher plants and tended to be relatively stable (Figure 2). In other words, compared to the lower plants, the higher plants contained more group A *AKR* genes. These suddenly amplified group A *AKR* genes might play an important role in the complex growth and development of higher plants.

Lastly, the divergence of selective pressure between the two groups of *AKR* genes was analyzed by the PAML4.9 program using ML codon models. In total, 12 out of 36 representative plants were selected in this analysis (Supplementary Table S3 and Figure S2). In *B. rapa*, the log-likelihood values of the *AKR* genes under the one-ratio and two-ratio models were lnL = −23476.932124 and −23474.846221, respectively (Supplementary Table S3). The likelihood ratio tests (LRTs) showed that the two-ratio model rejected the null model (one-ratio model), suggesting that the selective pressure differed significantly between the two groups (*p* < 0.05). In *B. rapa*, the ω (dn/ds) values for group A were lower than those in group B under the two-ratio model, indicating that group B *AKR* genes were under more relaxed selection constraints than group A *AKRs*. This factor might have led to group B *AKRs* having more diverse functions. Conversely, for *F. vesca*, group A *AKRs* were under more relaxed selection constraints (Supplementary Table S3). Interestingly, the number of *AKRs* in group A was greater than that in group B, suggesting that group A *AKRs* underwent more significant functional divergence. In addition, we found that the ω_1_/ω_0_ value gradually increased in these three plants (*P. patens*, *S. moellendorffii,* and *A. trichopoda*), indicating that selective pressure on group A *AKRs* also gradually increased. This result might explain the increase in the number of A genes. However, the two-ratio model did not apply to all plants, such as *M. truncatula* (*p* = 0.61) and *C. reinhardtii* (*p* = 0.36), indicating that the difference in the selective pressure between the two groups was not significant.

### 2.3. Copy Number Variation of AKR Homoeologous Genes Following WGT in B. rapa

**Figure 3:**
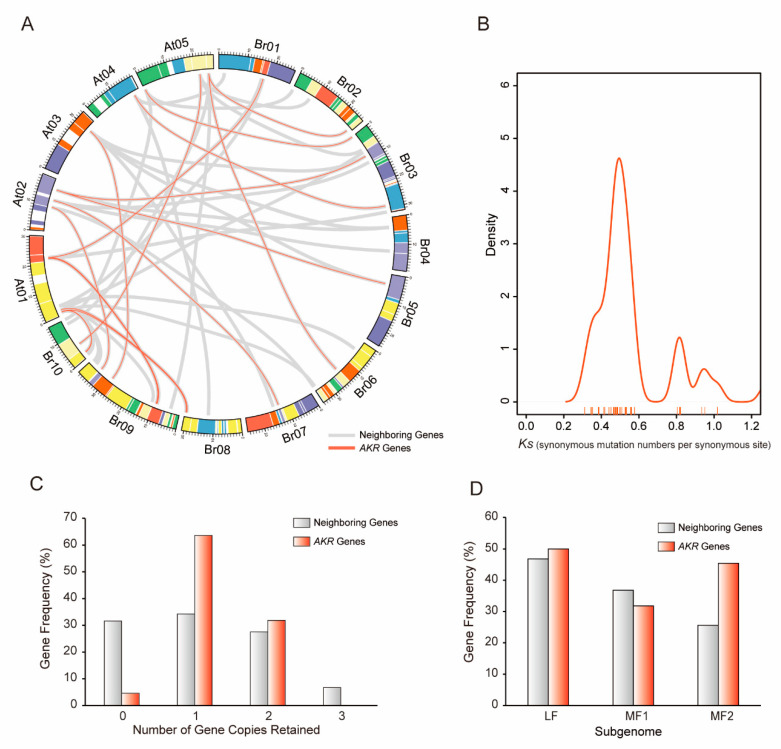
Retention of aldo-keto reductases (*AKR*) genes and their neighbor genes in the syntenic regions of *B. rapa* and *A. thaliana* and the *Ks* values of *AKR* syntenic gene pairs. (**A**) Collinear correlations of *AKR* genes and their neighbor genes in the *A. thaliana* and *B. rapa* genomes by Circos. (**B**) The distribution of *Ks* values for *AKR* syntenic gene pairs between *A. thaliana* and *B. rapa*. (**C**) Copy numbers of *AKR* genes and their neighbor genes after genome triplication and fractionation in *B. rapa*. (**D**) Retention of homolog copies of *AKR* genes and their neighbor genes in the three subgenomes (LF, MF1, and MF2) in *B. rapa*. LF: least fractionized; MF1: moderately fractionized; MF2: most fractionized.

The *B. rapa* genome underwent whole-genome triplication (WGT), making it a good material for studying gene duplication [17]. To investigate the copy number variation and degree of retention of *AKR* genes between *A. thaliana* and *B. rapa* during the *Brassica*-specific WGT event, first, the syntenic regions between them were identified using syntenic analysis with the MCScanX and BRAD databases. Here, we identified a total of 45 *B. rapa* regions syntenic to 22 *A. thaliana AKRs* (Figure 3A and Supplementary Figure S3A). In these regions, 25 out of 29 *B. rapa AKRs* were identified as syntenic gene pairs with *A. thaliana AKR* genes. The *Ks* (synonymous substitution rates) and *Ka* (nonsynonymous substitution rates) values were calculated for a total of 29 syntenic gene pairs. All ratios of *Ka*/*Ks* were less than one (Figure 3B), indicating that purifying selection occurred in the entire AKR gene family. Based on the *Ks* values, we speculated that the divergence of most *B. rapa AKR* genes relative to the *A. thaliana* orthologs occurred from 14 to 17 million years ago (MYA; Figure 3B and Supplementary Table S4), concurrent with the *Brassica*-specific WGT event (13–17 MYA) [17]. Specifically, partial *AKRs* experienced a duplication event from 27 MYA, ahead of the WGT event, suggesting their importance in plant growth and development.

Next, we compared the retention of *A. thaliana AKR* genes relative to the set of their neighboring genes (ten on either side, flanking the *AKR* genes) in *B. rapa* (Figure 3A,C). Only one (4.5%) *AKR* in *B. rapa* was completely lost, which was less than the loss in neighboring genes (~31%). Most *AKRs* were retained in one (63.6%) or two (31.8%) copies, which is significantly greater than the retention of the neighboring genes. No *AKR* was retained in three copies, but 6.7% of the neighboring genes were retained. The degree of retention between *AKR* and neighboring genes were varied among the three sub-genomes of *B. rapa*. Compared to the neighboring genes, more *AKR* homologs were retained in the MF2 sub-genomes (Figure 3A,D). This indicates that the homologs in MF2 contributed to the retention of *AKR* genes. Why did this sub-genome contain more homologs that were not consistent with those shown in a previous report by Wang et al. (2011) [17]? We further explored the gene duplication types of *AKR* genes to answer this question. All the syntenic gene pairs were identified non-randomly on the GBs (the conserved ancestral genomic blocks labeled A–X) between the *B. rapa* and *A. thaliana* chromosomes (Supplementary Figure S3A). By counting the homologs on the same GBs in *B. rapa*, 26.7% of the *AKR* genes were shown have experienced segmental duplication events (Supplementary Figure S3A). Furthermore, specific duplicated types of *AKR* genes and all genes in *B. rapa* were identified by the MCScanx program. We also noted that less segmental duplication was observable in *AKR* genes than at the whole genome level in *B. rapa* (Supplementary Figure S3B). Surprisingly, a great deal (37.9%) of tandem duplication was observable in the *AKR* genes, most of which were found in the MF2 sub-genome (Supplementary Table S5), which answers the previous question.

Lastly, the copy number variations of the two groups were analyzed in this family. Group A *AKR* genes were all retained, and one gene in group B was lost (Supplementary Figure S3C). However, two gene copies were found to be more plentiful in group A than in group B. Strangely, the number of *AKRs* in group B was greater than that in group A. This may be because ancestral group B genes were lost in *A. thaliana* or divergence occurred among group B genes during the WGT event in *B. rapa*. Overall, the *AKR* genes were preferentially retained but not significantly amplified. Although most *AKRs* were retained in *B. rapa*, many paralogs of *AKRs* were lost after polyploidization (Supplementary Figure S3C). Moreover, tandem duplication is the main reason for amplification of the *AKR* genes.

### 2.4. Structural Characteristics and Expression Pattern Analysis of AKR Homoeologous Genes in B. rapa and A. thaliana

**Figure 4:**
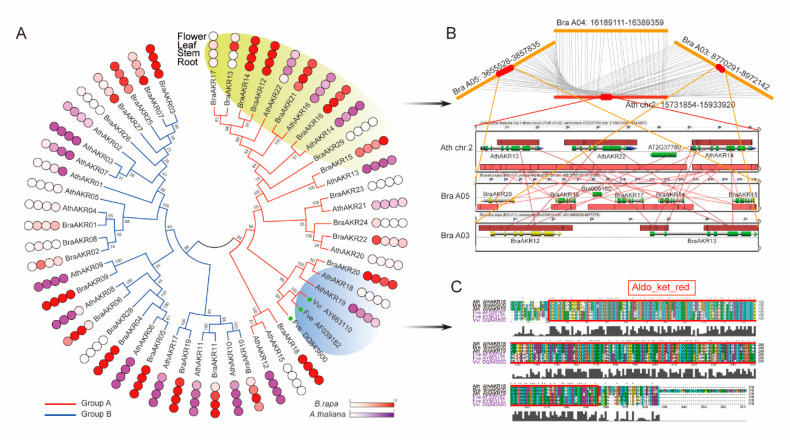
Expression patterns and evolutionary characteristics of aldo-keto reductases (*AKR*) genes in *A. thaliana* and *B. rapa*. (**A**) Phylogenetic relationship and expression pattern of AKRs in *A. thaliana* and *B. rapa*. The tissue expression analysis included root, stem, leaf, and flower. **(B**) Micro-syntenic analysis of part of the *AKR* genes in *A. thaliana* and *B. rapa* genome. (**C**) Multiple sequence alignments of GalUR orthologs.

To investigate the extent of the lineage-specific divergence of AKR proteins compared to the GalURs in *B. rapa* and *A. thaliana*, the phylogenetic relationships were reconstructed (Figure 4A and Supplementary Figure S4A). This reconstruction showed that the phylogenetic tree was also divided into A and B groups based on bootstrap values. GalURs belonged to group A and had a closer relationship to AthAKR18, AthAKR19, and BraAKR20 (Supplementary Figure S4A). Next, a total of 12 motifs were identified using the Multiple Expectation–Maximization for Motif Elicitation (MEME) program to discover the conservative motifs shared among the AKRs. The group A AKRs generally shared similar motif compositions, except for BraAKR29, which lost most of its motifs. The group B AKRs revealed an obvious divergence among the subclasses. The AKR proteins clustered into the same subclass generally shared similar motif compositions (Supplementary Figure S4B). The gene structures were also analyzed among these AKR genes. Overall, there were no significant divergences between group A and B (Supplementary Figure S4C), while similar gene structures were found within the same subclasses, especially within the homologs on the same branch of the phylogenetic tree.

Subsequently, to detect the functional divergence of the *AKR* genes (mainly among the homologs), we further explored their expression in different tissues (roots, stems, leaves, and flowers) in *B. rapa* and *A. thaliana* (Supplementary Table S6 and S7). Overall, nearly half of the genes had high expression levels in all tissues. The expression levels of the 12 genes (*BraAKR08/17/23/24/26/28/29* and *AthAKR04/05/15/18/20*) were not detected in each tissue, and the rest of the genes had high expression levels in specific tissues (Figure 4A). All 10 pairs of orthologs were detected to have similar expression levels, and the rest showed a functional divergence. There were also obvious differences in the expression levels among paralogs (Figure 4A). To further explore the relationship between gene duplication and expression level, we selected a typical region in group A. Both segmental and tandem duplication were found among these genes by microsynteny analysis using CoGe (Figure 4B). Interestingly, these genes were present on a large branch of group A (Figure 4A), indicating their closely phylogenetic relationship. Furthermore, most homologs were found to have similar expression levels (Figure 4A).

Lastly, we focused on the GalUR orthologs. AthAKR18, AthAKR19 and BraAKR20 were highly homologous to the three known GalURs (AF039182, AY663110, and DQ843600) (Figure 4A). The protein sequences were very similar, especially Aldo_ket_red domain sequences (Figure 4C). The two paralogs in *A. thaliana* were found to have divergent expressions, while *AthAKR19* and its ortholog *BraAKR20* had similar expression levels.

### 2.5. Cloning and Identification of Group A AKR Genes in Pak-choi

The results of the evolutionary history and sequence characteristics suggest that group A *AKRs* are more conservative and can be considered as *GalUR* candidate genes. To further explore their functions, all 12 genes were cloned in Pak-choi based on specific primers designed according to their orthologs in *B. rapa*. According to detection using the SMART tool, the deduced AKR proteins all contained the Aldo_ket_red domain. Then, these cloned *AKR* genes were named *BrcAKRs*, and their serial numbers were assigned according to their orthologs in *B. rapa* (Supplementary Table S8).

To further explore the phylogenetic relationships of these 12 cloned genes, a new phylogenetic tree was reconstructed with all the AKR proteins of *B. rapa* and *A. thaliana*. This tree clearly forms two groups, and all the AKRs in Pak-choi were assigned to group A, showing a visible orthologous relationship with *B. rapa* (Supplementary Figure S5A). Next, we established a 3D (three-delimitation) structural model of the BrcAKRs using the ConSurf web server based on a multiple sequence alignment of the group A AKR proteins among *A. thaliana*, *B. rapa,* and Pak-choi to better understand the conserved amino acid distributions and the conserved spatial structural evolution of group A AKR proteins in Pak-choi (Supplementary Figure S5B). The amino acids were represented by different colored balls divided into a discrete scale of nine grades (one for the most variable positions and nine for the most conserved positions) for visualization. According to the putative structural model of the BrcAKR protein, the inner part of group A proteins were conserved and the N- and C-terminal parts were divergent, indicating that the Aldo_ket_red domain was very conserved.

### 2.6. Expression of BrcAKRs and AsA Content under Multiple-Stress Treatments in Pak-choi

In previous reports, light was shown to exert a significant impact on changes to the AsA content in plants [18]. At the same time, it has been reported that the transcription of *AKR* genes can be induced under strong light conditions [19,20]. Thus, to explore the light-mediated regulation of AsA and *AKR* gene expression in Pak-choi, 48 h of continuous light and dark treatments were used. A clear upward trend in AsA content was found in Pak-choi exposed to continuous light for 48 h. In turn, after 48 h in darkness, the AsA levels in leaves continued to decline (Supplementary Figure S6). For *AKR* genes, a significant divergence in expression levels was found after these dark–light treatments. Under light treatments, the transcription levels of these genes exhibited a certain upward trend at the beginning but were significantly down-regulated at 48 h, except for *BrcAKR22*. In addition, five genes *(BrcAKR12/14/15/20/21)* showed the highest expression levels at 12 h (Supplementary Figure S6). Under dark treatment, differences also emerged in the expression patterns among these genes. The transcript levels of most genes were up-regulated at different time points, except for *BrcAKR14*, *17*, *18,* and *21* (Supplementary Figure S6). In summary, although these genes showed varying degrees of responses induced under light or dark treatments, we did not find a clear linear relationship between those responses and AsA content.

**Figure 5:**
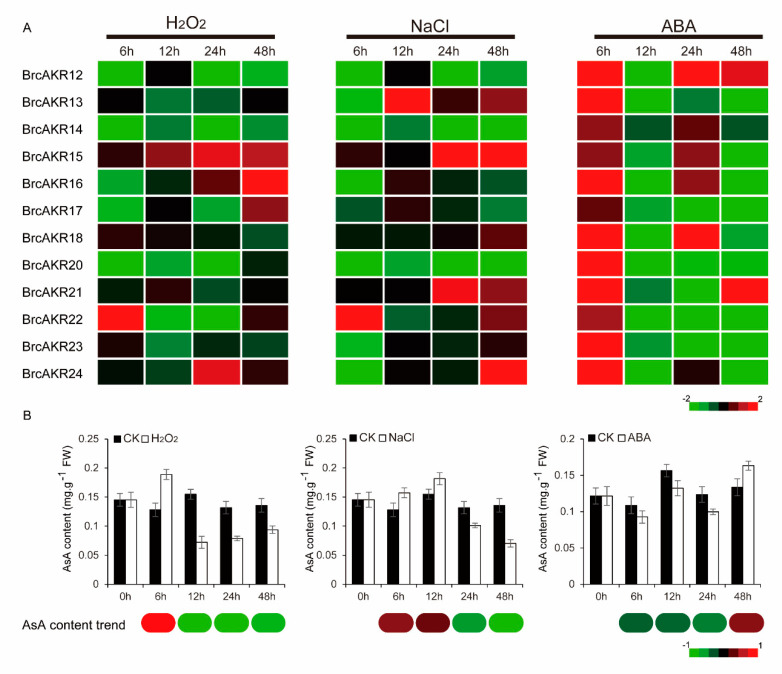
Expression of aldo-keto reductases (*AKR*) genes and ascorbic acid (AsA) content level under NaCl, H_2_O_2_, and abscisic acid (ABA) treatments in Pak-choi leaves. (**A**) Transcript level analysis of *AKR* genes. (**B**) AsA content level. The bar at the bottom of each heat map represents the relative values.

We also investigated the relationship between the expression patterns of *GalUR* candidate genes and AsA content under multiple stress treatments, including H_2_O_2_, NaCl, and abscisic acid (ABA). These stress treatments can all induce oxidative stress directly or indirectly and change the AsA content in plants [2]. The expression patterns of these *GalUR* candidate genes showed divergent responses to these stresses, and most genes were strongly induced at different time points (Figure 5A). In particular, the transcript levels of all genes were significantly up-regulated at 6 h under ABA stress. One possible reason for this result is that *AKRs* might participate in the regulation of NADPH oxidase activity in ABA signal transduction [13]. Under H_2_O_2_ and NaCl stresses, the expression trend was relatively complex, and the transcript levels of most genes were reduced. In detail, the expression levels of most genes did not show a clear upward trend at the beginning, except for *BrcAKR22*. However, the transcription of some genes was activated as time progressed, including *BrcAKR13*, *15*, *21,* and *24* (Figure 5A). Overall, the *AKR* genes were more complex in response to multiple stresses, and homologous genes manifested a significant functional divergence. Meanwhile, the AsA content was also measured under these stresses. Under H_2_O_2_ stress, the AsA content increased at 6 h and then decreased. Under NaCl stress, the levels increased prior to 12 h and then decreased. Under ABA stress, the AsA content continuously decreased until 48 h, at which point they significantly increased (Figure 5B). Only the expression pattern of *BrcAKR22* was consistent with the changes in AsA content under H_2_O_2_ and NaCl stress (Figure 5).

**Figure 6:**
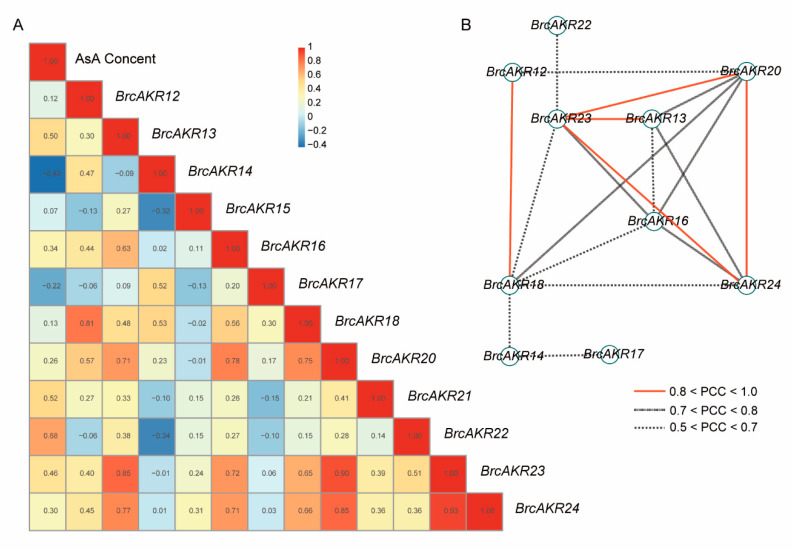
Effect of light on the ascorbic acid (AsA) level and the expression patterns of aldo-keto reductases (*AKR*) genes in Pak-choi leaves. (**A**) The bar chart indicates the AsA levels. (**B**) Line charts: transcript levels of *AKR* genes. For each sample, the transcript levels were normalized with those of actin (control). Data are the mean values ± SD of three individual experiments (*n* = 3).

To clearly understand the connection between the transcript levels of *AKR* genes and AsA content, the Pearson correlation coefficients (PCCs) were established between the AsA content and the relative expression trends of the genes. Three genes, including *BrcAKR13*, *21,* and *22,* appeared to have a positive correlation (PCC > 0.5) with AsA content, particularly *BrcAKR22*, which had a PCC of 0.68 (Figure 6A). Through this co-expression relationship, the divergences between homologous genes were further clarified (Figure 6A).

Lastly, all PCCs with a 0.05 (*p*-value) significance level were collected and visualized by the Cytoscape program to construct stress co-expression networks for these genes in Pak-choi. All 19 edges (regulatory relationship) and 10 nodes (genes) were included in this network. Interestingly, *BrcAKR23* was co-expressed simultaneously with three genes under these stresses (Figure 6B), indicating its important role in central regulation.

## 3. Discussion

The AKR superfamily is commonly found in bacteria, archaebacteria, yeast, animals, and plants. One *AKR* was first identified to encode GalUR in a strawberry and was proven to play an important role in AsA biosynthesis [5,21,22]. Subsequently, GalUR homologs were revealed to be involved in the biosynthesis of AsA from _d_-galacturonic acid in grapes and oranges [9,19]. However, the evolutionary patterns of *AKR* genes in plant have not yet been studied in depth, especially their phylogenetic relationship with *GalUR* genes.

In this study, all 1268 *AKR* genes were identified in the 36 selected plants to explore the evolutionary patterns of the AKR family in plants and the relationship of *AKRs* with *GalUR* genes. we found that *AKR* genes might be involved in physiological activities starting with algae. Previous reports confirmed that *AKR* genes indeed played important roles in plant growth and development, especially for the detoxification of xenobiotics in algae [23]. It was also confirmed that these antioxidants were abundant in algae and prokaryotic cyanobacteria, which were often used as phytonutrients [24]. GalUR, an enzyme encoded by one member of the AKR gene family, was purified to homogeneity from *Euglena gracilis* and could be activated by H_2_O_2_. However, no obvious feedback regulation of this enzyme’s activity by AsA was observed [25], suggesting that GalUR homologs have existed since algae, although their function in AsA biosynthesis remains unclear. In addition, the gene structures of *AKRs* showed significant divergences in algae. The exon number, moreover, is around five in bryophytes and lycophytes (even in angiosperms), suggesting that the origin and differentiation of *AKRs* in plants begin with algae. In land plants, more complicated organ systems and structures might require more complicated gene structures of *AKRs* to maintain their biological functions.

The AKRs were divided into two distinct groups, groups A and B (Figure 1A,B). Compared to the group B AKRs, group A AKRs gradually increased from algae to *A. trichopoda* (basal angiosperm). This is possibly a functional requirement since land plants require more complex environmental adaptability than algae. This process caused group A *AKR* genes, such as *AKR4C9* and *GalUR*, to obtain greater neofunctionalization. These genes have been confirmed to play important roles in plant developmental processes, especially in resisting the multiple stresses in angiosperms [13,14,22]. Based on a selective pressure analysis, group A *AKRs* experienced increasingly more relaxed selection constraints from bryophytes to basal angiosperm. During this period, group A *AKRs* could have experienced positive selection, which allowed new duplicate genes to branch into different functions. Thus, the functional requirements could explain the rapid expansion of group A *AKRs* before the origin of the angiosperms. However, the genome evolution of angiosperms is characterized by polyploidization, which is typically accompanied by different gene duplications [26]. The rapid expansion of group A *AKRs* and all *AKRs* was diversified in different plant species. Different species have different growth environments and morphological developments. Different species also experience different whole-genome duplication (WGD) or whole-genome triplication (WGT) events.

For the *Brassica* species, our findings further suggest that the *AKR* genes had a high degree of retention following WGT. Only one homolog (group B gene) was lost during the divergence between *B. rapa* and *A. thaliana* from the last common ancestor, and no homolog was lost in *B. oleracea* (Supplementary Table S5). This is consistent with the gene balance hypothesis [27], suggesting that *AKR* genes are involved in important metabolic activities. However, not all homologs were retained as three copies during the *Brassica*-specific WGT event, and many paralogs were lost. In addition, tandem duplication was a major factor responsible for the expansion of the AKR gene family in *B. rapa*, similar to *A. thaliana* (Supplementary Table S5). This is also similar to other gene families, such as PG (polygalacturonase) and GST (glutathione S-transferase) in *Populus* [28,29]. Furthermore, four *B. rapa AKRs* (all group B genes) were not identified in the syntenic regions with *A. thaliana*, indicating that group B *AKR* genes developed a new independent branch during the *Brassica*-specific WGT event. The phylogenetic analysis among the AKRs in *B. rapa* and *A. thaliana* also confirmed this. In general, WGD events were typically followed by substantial gene loss [30]. Gene loss in the AKR gene family might reflect the general evolutionary patterns of large gene family evolution [31]. In addition, we explored *AKR* homoeologous genes in the amphidiploid species *B. napus* (AC genome, *n* = 19) via hybridization from *B. rapa* (A genome, *n* = 10) and *B. oleracea* (C genome, *n* = 9) [32]. This analysis further confirmed that *AKR* homoeologous genes use divergence in different species to adapt to complex environments. Compared to group B AKRs, group A AKRs showed better homology (Supplementary Figure S7). The duplication, divergence, and loss events in the AKR gene family are considered an important mechanism underlying the generation of genomic diversity among eukaryotic species [33].

Through analyses of (i) gene retention, (ii) nucleotide distance, (iii) protein structure, and (iv) selection pressure, we found that the AKRs in group A were more conservative than those in group B. Group A AKRs showed high similarity (93.44% gene pairs e-value < 1 × e^−20^ and 98.96% gene pairs e-value < 1 × e^−10^) with FaGalUR according to the analysis using BLASTP, suggesting that group A genes are candidate GalURs. We cloned and sequenced 12 group A *AKR* genes from the cDNA libraries of stress-induced Pak-choi, and verified the relationships between their expression patterns and AsA content under multiple stresses. These genes are highly homologous to those in *B. rapa* and *A. thaliana*. The structural protein model shows that the N- and C-terminal parts of AKR proteins are divergent, while the Aldo_ket_red domain parts are conservative. Previous reports have indicated that AKR proteins and AsA are important active substances and antioxidants involved in plant growth and development or resisting multiple stresses [13,34]. *VvGalUR,* one of the *AKR* genes in grapes, was confirmed to be induced under high levels of light to increase the AsA content in fruits [19]. The *A. thaliana* AKR4C9 enzyme can also be induced under strong light to detoxify sugar-derived reactive carbonyls [20]. In addition, AsA content was significantly increased in plants exposed to strong light [18]. However, we did not acquire enough evidence to prove that there is a clear linear relationship between the activity of *AKR* homologs and AsA content under strong light conditions in Pak-choi. Nevertheless, some *AKR* homologs indeed showed different degrees of response under strong light treatments. The heterologous overexpression of *FaGalUR* in potato not only increased AsA content but also induced tolerance to various abiotic stresses [21]. *SlAKR4B*, an ortholog to *FaGalUR*, encodes a functional enzyme in tomato and is involved in the stress response but is not strongly correlated with AsA content [12]. The well-characterized *AKR4C9* has also been found to be strongly induced under different abiotic stress factors [14,35]. In this study, *AKR* genes were also found in response to multiple stresses. Surprisingly, the expression trend of *BrcAKR22* was found to have a significant correlation with changes in AsA content, especially under NaCl and H_2_O_2_ treatments. We speculated that this gene might be involved in the galacturonate pathway. The enzyme activity of *GalUR* might differ according to the plant stress response. Further studies should be conducted to verify *GalUR*’s substrate and clarify its mechanism. *BrcAKR23* showed similar expression trends with the three homologs. Moreover, we found an observable divergence in the expression of *AKR* paralogs under multiple treatments in Pak-choi. This result confirmed our doubts that the neofunctionalization or subfunctionalization models apply to these duplicated genes.

**Figure 7:**
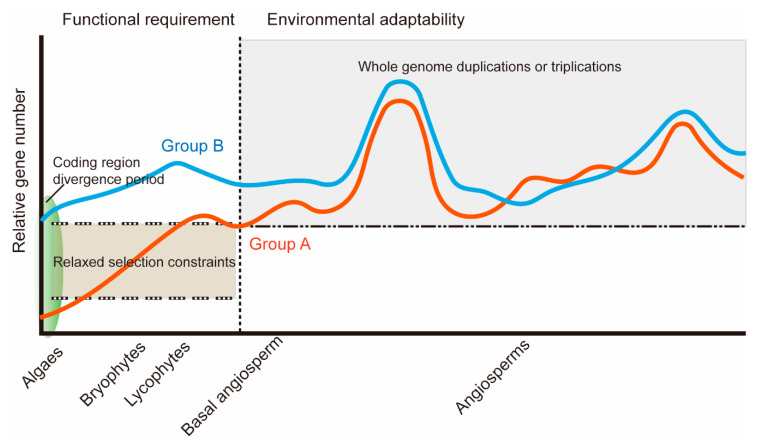
Proposed evolutionary model of aldo-keto reductases (*AKR*) genes in the plant kingdom. The orange line represents group A *AKR* genes, and the blue line represents group B *AKR* genes. The green oval represents the *AKR* gene coding region divergence period.

Overall, we determined the evolutionary pattern of *AKR* genes in plants (Figure 7). The importance of *AKR* genes, even *GalUR*, can be traced back to algae; the gene coding regions of *AKRs* began to differentiate during this period. Subsequently, there was a clear amplification of the AKR gene family, especially group A, from bryophytes to basal angiosperm due to functional requirements. As a result of the different WGD or WGT events and different levels of loss and retention, the quantities of the two groups of *AKR* genes are species-specific in angiosperms. At the same time, the neofunctionalization and subfunctionalization models applied randomly to duplicated genes, and the gene co-expression network were also found in the large gene family in response to complex natural environments.

## 4. Materials and Methods

### 4.1. Identification of AKR Genes in Comparison Species

All files related to the sequence data for the 36 genomes selected in this study were downloaded from the relevant databases and Phytozome (see in Supplementary Table S1). The Hidden Markov Model (HMM) profiles of Aldo-ket_red (PF00248) were obtained from the Pfam database (http://pfam.xfam.org/). Hmmsearch, which is part of the HMM software package, was used to identify the putative AKR proteins with the best domain e-value cutoff of 1e^−4^. Then, the potential sequences were analyzed using SMART (http://smart.embl-heidelberg.de/), Pfam (http://pfam.xfam.org/), and the NCBI database (http://www.ncbi.nlm.nih.gov/). The FGENESH program was used to rectify the incorrect coding regions of these genes [36]. Finally, an in-house Perl program was used to obtain basic information on the AKR sequences.

### 4.2. Phylogenetic and Molecular Evolution and Orthologous Analysis of AKR Gene Family

For the phylogenetic analysis, the MUSCLE program was first used to align the full-length protein sequences of the AKRs with default parameters [37]. Then, the MEGA 6.0 program was used to construct a phylogenetic tree via the ML method, which was ultimately examined by the NJ and MP methods [38]. The nucleotide divergence between sequences was also estimated by MEGA 6.0 using the Jukes–Cantor model with the bootstrap value set to 1000 replications.

The variations in selective pressure between the group A and B *AKR* genes were evaluated by PAML according to the method by Yang and Nielsen (2000) [39]. In total, 12 out of the 36 plant species, including *Fragaria vesca*, *Medicago truncatula*, *Brassica rapa*, *Arabidopsis thaliana*, *Citrus sinensis*, *Solanum lycopersicum*, *Vitis vinifera*, *Oryza sativa*, *Amborella trichopoda*, *Selaginella moellendorffii*, *Physcomitrella patens,* and *Chlamydomonas reinhardtii*, were used to measure the selective pressure values. The branch models of CODEML in PAML were used to estimate ω = (dN/dS). The specific methods are outlined in our previous paper [40].

The OrthoMCL program was used to identify the homologous *AKR* genes among the *A. thaliana* and three *Brassica* species (*B. rapa, B. oleracea,* and *B. napus*) [41]. Then, the MCL clustering algorithm was used to deduce the relationship between these homologous genes. The ClusterVenn (https://orthovenn2.bioinfotoolkits.net/cluster-venn) program was used to produce the AKR ortholog groups, and the Cytoscape software was used to build the network of the AKR relationships [42].

### 4.3. Synteny and Ks Analysis of AKR Genes Between A. thaliana and B. rapa

The McScanX program (http://chibba.pgml.uga.edu/mcscan2/) was used to construct the synteny within and between the genome (the parameters are based on those used in our previous paper [43]). The duplicate_gene_classifer program, which incorporates the MCScanX algorithm, was used to identify the potential duplicate genes [44].

The positions of the *AKR* genes on the conserved collinear blocks were verified by searching for homologous genes among *A. thaliana* and three sub-genomes (LF: least fractionized, MF1: moderately fractionized, and MF2: most fractionized) of the *Brassicas* species at BRAD (http://brassicadb.org/brad/searchSynteny.php). The Circos software was used to draw a syntenic diagram [45]. The conservation of chromosomal synteny around the *AKR* genes in *A. thaliana* and *B. rapa* were derived from the CoGe program (http://www.genomevolution.org/CoGe/GEvo.pl).

The *Ks* and *Ka* values were obtained by processing the syntenic gene pairs between *B. rapa* and *A. thaliana.* Firstly, the MUSCLE program was used for the protein sequence alignment [37]. Then, an in-house Perl script based on ParaAT was used to back-translate the protein alignments into coding-sequence alignments [46]. Finally, based on the coding-sequence alignments, the *Ks* values were calculated using the method of Nei and Gojobori via the KaKs_calculator [47]. The *Ks* values were then plotted as histograms using the R program [48]. The divergence time was calculated with the formula T = *Ks*/2R [49].

### 4.4. Motif Identification and the Exon–Intron Structural Analysis of AKR Genes

The online Multiple Expectation–Maximization for Motif Elicitation (MEME version 5.0.0) program was used to identify and analyze the conserved motifs of the AKR proteins of *B. rapa* and *A. thaliana* using default parameters, except for the following: the maximum number of motifs was set to 12, and the optimum motif width was set to ≥10 and ≤100.

An in-house Perl script was used to parse the gene-structure information of the *AKR* genes from the General Feature Format (GFF) files of *B. rapa* and the *A. thaliana* genome. Then, the online program GSDS (http://gsds.cbi.pku.edu.cn/) was used to draw the exon–intron structures.

### 4.5. Expression-Pattern Analysis of AKRs in B. rapa and A. thaliana

The Illumina RNA-seq data previously generated by Tong et al. (2013) [50] was used for the expression profiling of *AKRs* in *B. rapa.* The AtGenExpress Visualization Tool (AVT) with mean-normalized values was used for the expression profiling of the *AKRs* in *A. thaliana* [51]. Four tissues, including the root, stem, leaf, and flower, were analyzed, and the R program was used to draw the heatmaps [48].

### 4.6. Plant Materials, Growth Conditions, Stress Treatments, and Statistics

Pak-choi (*B. rapa* ssp. *chinensis* cv. *suzhouqing*) was used in this study. The seedlings were soaked in distilled water and germinated on moist filter paper in darkness at 24 °C for 2 days. The germinated seeds were then transferred to pots containing soil and a vermiculite mixture (3:1) and cultivated in a greenhouse whose controlled-environment growth chamber was programmed for 75% humidity, light (100 mmol m^−2^ s^−1^) 16 h/25 °C and dark 8 h/20 °C. Five-leaf-old seedlings were used for the subsequent experiments. (i) Light/darkness treatment: based on our previous study [52], the samples were collected at 0, 3, 6, 12, 24, and 48 h. (ii) Multiple-stress treatments: some plants were transferred to plastic containers with 1/2 Hoagland solution (pH 6.5) [53]. The controlled-environmental growth chamber was the same as before. After 1 week of acclimatization, the plants were challenged with H_2_O_2_, ABA, and salt treatments. The other conditions were left unchanged, and the concentration of the solution was maintained with 10 mM H_2_O_2_, 100 μM ABA, and 100 μM NaCl. The treatments also ran over a continuous time course of 0, 6, 12, and 24 h. All treatments were performed with three biological replicates, and all samples were frozen in liquid nitrogen and stored at −70 °C until further analysis.

The total RNA was isolated from 0.1 g of frozen Pak-choi leaf tissues under multiple treatments using an RNA kit (RNAsimply total RNA Kit, Tiangen, Beijing, China) according to the manufacturer’s instructions. After assessing the quality and quantity of each RNA sample, *BrcAKRs* were cloned according to the gene-specific primers (Supplementary Table S9) that were designed based on the coding sequence (CDS) sequences of Chinese cabbage *AKR* orthologs. Then, based on the cloned sequences, we designed anther gene-specific primers for qRT-PCR analysis (Supplementary Table S9). The qRT-PCR reactions were performed using a 7500 Fast Real-Time PCR System (Applied Biosystems) with at least three biological replicates according to our previous study [52]. The Ct value method was used to calculate the relative expression ratio of each gene [54], and *Actin* (GenBank: AF111812) was used as an internal control to normalize the expression levels of the target genes among the different samples.

The AsA levels of each sample were analyzed according to the procedure described in our previous study [52]. HPLC assays at a wavelength of 245 nm were used to measure the AsA content. Finally, the Pearson correlation coefficients (PCCs) between the AsA content and the transcript levels of the *BrcAKRs* were calculated using our in-house Perl script. The PCCs were calculated according to their relative expression trends at different treatment times, as previously described [55,56]. The R package was then used to analyze the correlation [48]. The gene pairs whose PCCs were significant at a 0.05 significance level (*p*-value) were collected for co-expressed networks using the Cytoscape program (version 3.1) [42].

## Supplementary Materials

Supplementary Materials can be found at https://www.mdpi.com/1422-0067/21/17/5987/s1. Figure S1. Distribution of number statistics of aldo-keto reductase (*AKR*) genes in plant species. Figure S2. Phylogenetic trees of aldo-keto reductase (*AKR*) proteins of the selected plant species for PAML analysis. Figure S3. Aldo-keto reductase (*AKR*) homologous genes in segmental syntenic regions of the *Brassica rapa* and *Arabidopsis thaliana* genomes and their duplication types in *B. rapa*. Figure S4 An analytical view of the aldo-keto reductase (*AKR*) gene family in *Brassica rapa* and *Arabidopsis thaliana*. Figure S5. Phylogenetic tree of cloned aldo-keto reductase (*AKR*) genes and conserved evolution of AKR-3D. Figure S6. Effect of light on the ascorbic acid (AsA) level and the expression patterns of aldo-keto reductase (*AKR*) genes in Pak-choi leaves. Figure S7 Relationship of aldo-keto reductase (*AKR*) homologous genes among *Arabidopsis thaliana*, *Brassica rapa*, *Brassica oleracea* and *Brassica napus*. Table S1. The database source of the selected plant genome. Table S2. The information of aldo-keto reductase (*AKR*) gene family in the selected 36 plant species. Table S3. Summary statistics for detection of selection using branch-specific models of PAML. Table S4. Pairwise comparison of *Ka* and *Ks* values about aldo-keto reductase (*AKR*) gene pairs between *Arabidopsis thaliana* and *Brassica rapa*. Table S5. Identification of aldo-keto reductase (*AKR*) homologous genes among *Arabidopsis thaliana* and three subgenomes in *Brassicas* species. Table S6. The FPKM values of the aldo-keto reductase (*AKR*) genes in *Brassica rapa*. Table S7. The normalized values of the aldo-keto reductase (*AKR*) genes in *Arabidopsis thaliana*. Table S8. *BrcAKR* genes in Pak-choi (*Brassica rapa* ssp. *chinensis*). Table S9. Primers for *BrcAKR* genes cloning and quantitative real-time PCR.
