# Supplementary material for: Molecular Evolutionary and Expression Pattern Analysis of AKR Genes Shed New Light on GalUR Functional Characteristics in Brassica rapa
Source: Int J Mol Sci. 2020 Aug 20;21(17):5987. doi: 10.3390/ijms21175987 (PMC7503288; doi:10.3390/ijms21175987)
Supplement: Supplementary file 1 [file ijms-21-05987-s001.zip › Supplementary Figures.pdf]

International Journal of *Molecular Sciences*

MDPI

Supplementary Material

Molecular Evolutionary and Expression Pattern Analysis of *AKR* Genes Shed New Light on *GalUR* Functional Characteristics in *Brassica rapa*

Weike Duan [1,2], Zhinan Huang [1,*], Ying Li [2,*], Xiaoming Song [3], Xiaochuan Sun [1], Cong Jin [1], Yunpeng Wang [1] and Jizhong Wang [1]

1 College of Life Sciences and Food Engineering, Huaiyin Institute of Technology, Huai'an 223003, China; weikeduan@126.com (W.D.); huangzn@hyit.edu.cn (Z.H.); sunxch@hyit.edu.cn (X.S.); jincong@hyit.edu.cn (C.J.); ypwang@hyit.edu.cn (Y.W.); hgxjz@hyit.edu.cn (J.W.)

2 State Key Laboratory of Crop Genetics and Germplasm Enhancement, Key Laboratory of Biology and Germplasm Enhancement of Horticultural Crops in East China, College of Horticulture of Nanjing Agricultural University, Nanjing 210095, China; yingli@njau.edu.cn

3 School of Life Science and Center for Genomics and Computational Biology, North China University of Science and Technology, Tangshan 063210, China; songxm@ncst.edu.cn

* Correspondence: huangzn@hyit.edu.cn (Z.H.); yingli@njau.edu.cn (Y.L.); Tel.: +86-0517-8355-9216 (Z.H.); Tel.: +86-025-8439-5756 (Y.L.)

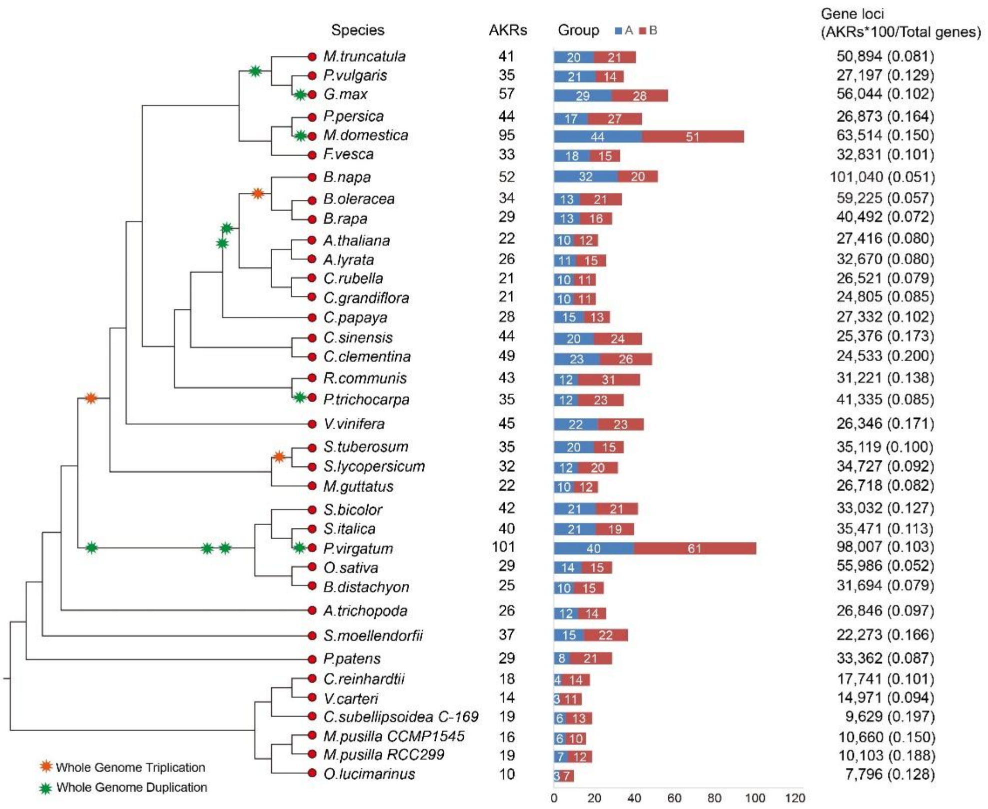

Figure S1. Distribution of number statistics of aldo-keto reductase (*AKR*) genes in plant species.

From left to right: (i) phylogenetic relationship of all selected species; (ii) the numbers of *AKR* genes in every species; (iii) the numbers of group A and B *AKR* genes; (iv) the percentage of *AKR* genes occupy all genes of every species.

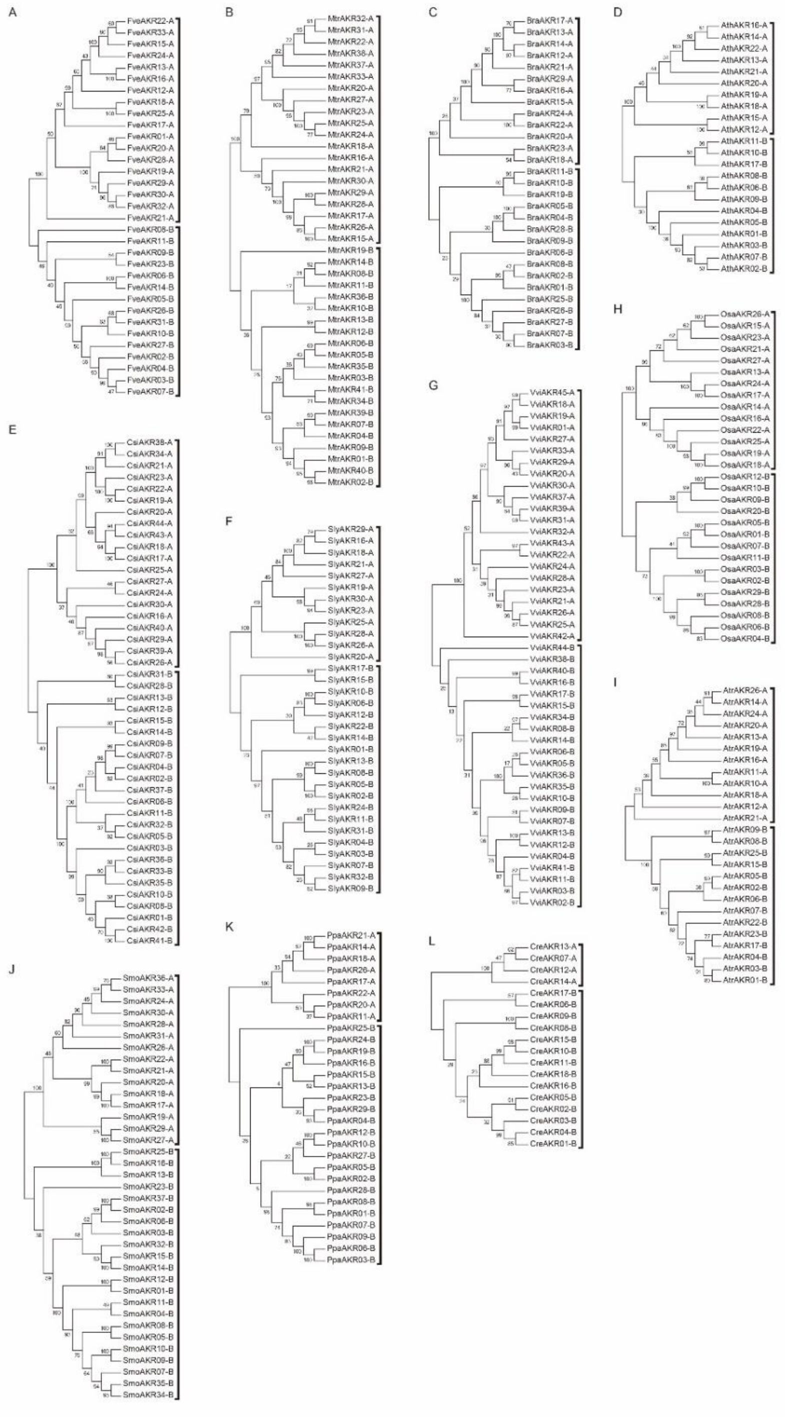

Figure S2. Phylogenetic trees of aldo-keto reductase (AKR) proteins of the selected plant species for PAML analysis

A: *Fragaria vesca*; B: *Medicago truncatula*; C: *Brassica rapa*; D: *Arabidopsis thaliana*; E: *Citrus sinensis*; F: *Solanum lycopersicum*; G: *Vitis vinifera*; H: *Oryza sativa*; I: *Amborella trichopoda*; J: *Selaginella moellendorffii*; K: *Physcomitrella patens*; L: *Chlamydomonas reinhardtii*.

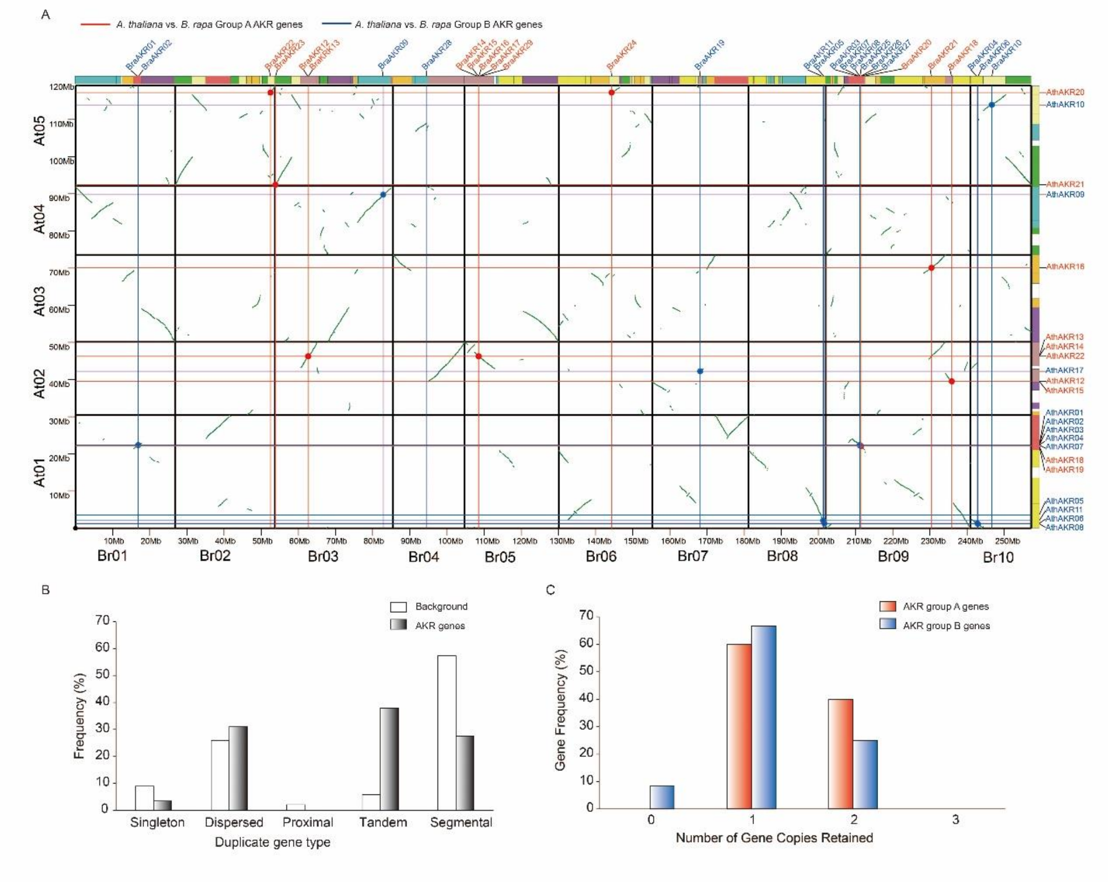

Figure S3. Aldo-keto reductase (*AKR*) homologous genes in segmental syntenic regions of the *Brassica rapa* and *Arabidopsis thaliana* genomes and their duplication types in *B. rapa*. (A) Conserved collinear blocks of genes (green irregular lines) are shown between the 10 *B. rapa* chromosomes (horizontal axis) and the five *A. thaliana* chromosomes (vertical axis). Red and green dots indicate group A and B *AKR* homologs in the two species. (B)The different duplicated types (singleton, dispersed, proximal, tandem and segmental) were counted in *B. rapa*. Open boxes indicate the whole genome level. (C) Copy numbers of group A and B *AKRs* after genome triplication and fractionation in *B. rapa*.

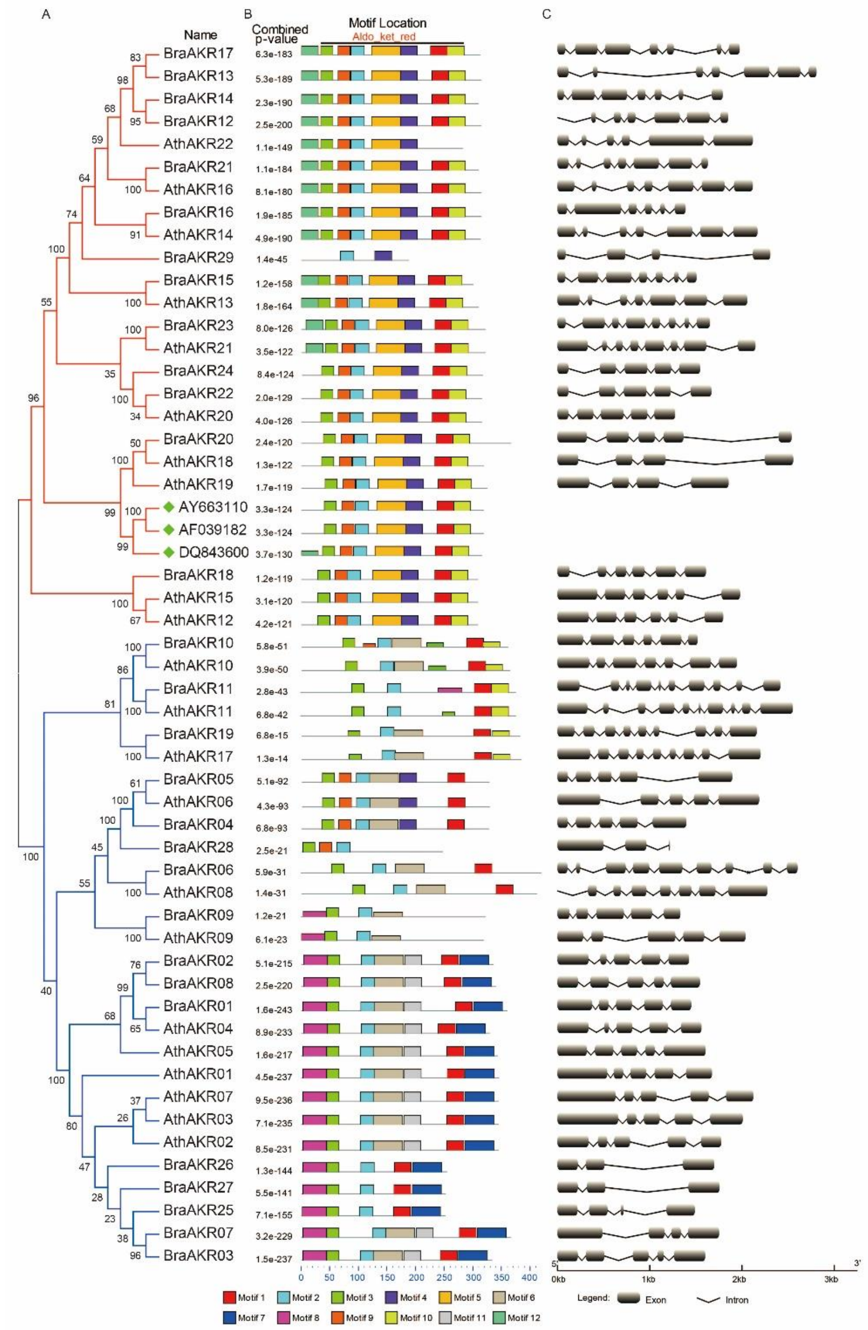

Figure S4 An analytical view of the aldo-keto reductase (AKR) gene family in *Brassica rapa* and *Arabidopsis thaliana*.

(A) Protein maximum-likelihood (ML) tree: this tree was constructed using the ML method, and bootstrap values were calculated with 1000 replications. Group A AKRs are colored red and group B AKRs are colored blue. (B) Protein structure: the search for the common motifs shared among the AKRs proteins of each group was performed using MEME; the clade situation was in the bottom. (C) Gene structure: the exons are highlighted by black boxes; introns are shown as broken lines.

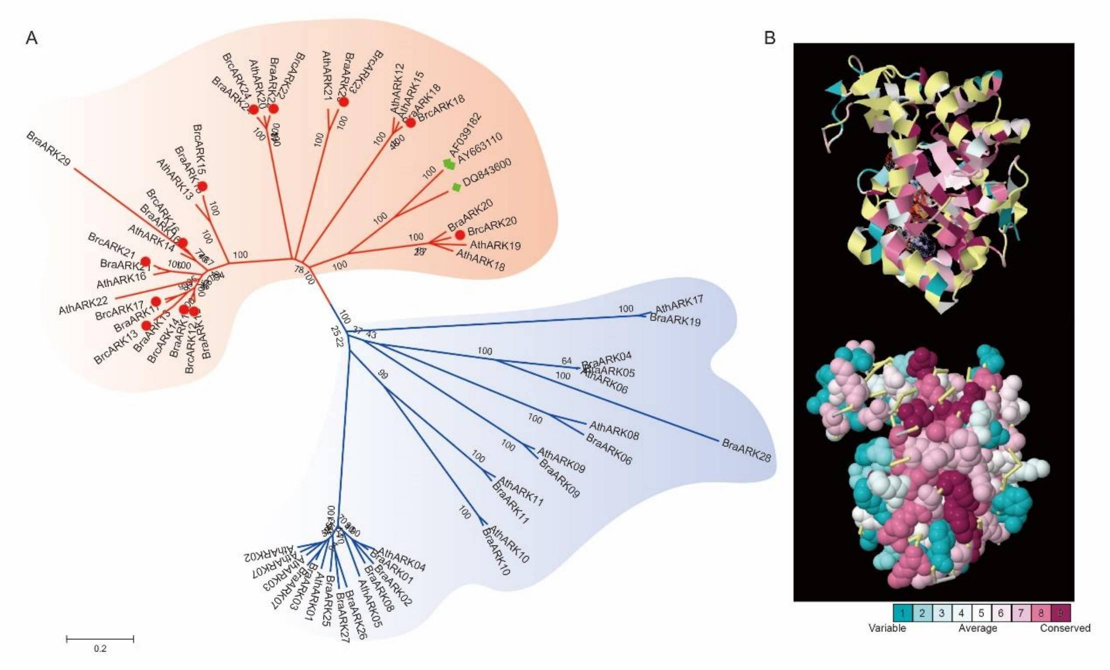

Figure S5. Phylogenetic tree of cloned aldo-keto reductase (*AKR*) genes and conserved evolution of AKR-3D.

(A) Phylogenetic tree among cloned *AKR* genes and *A. thaliana* and *B. rapa AKR* genes. Red represents the cloned genes and green represents GalUR genes. (B) Crystal structure of the putative AKR protein based on PDB:3H7U model. Residues are colored according to their levels of conservation. The score is 1 to 9 as blue to purple, which represents viable to conserve.

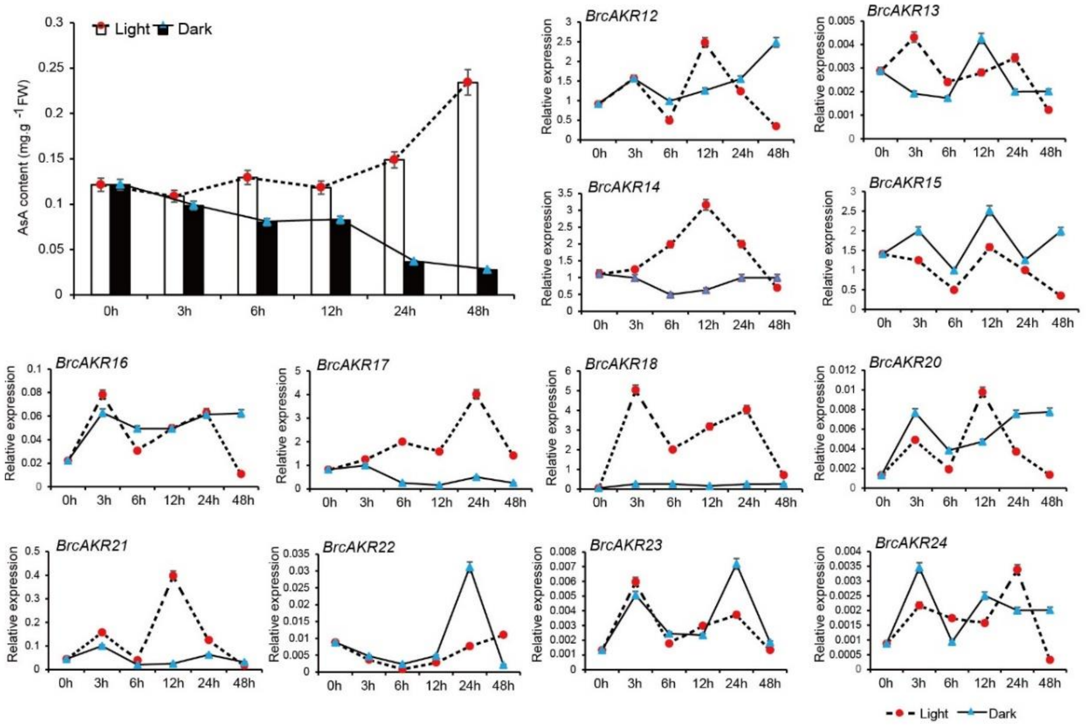

Figure S6. Effect of light on the ascorbic acid (AsA) level and the expression patterns of aldo-keto reductase (*AKR*) genes in Pak-Choi leaves.

Bar chart indicate AsA levels. line charts: Transcript levels of *AKR* genes. For each sample, transcript levels were normalized with those of actin (control). Data are the mean values ± SD of three individual experiments (n = 3).

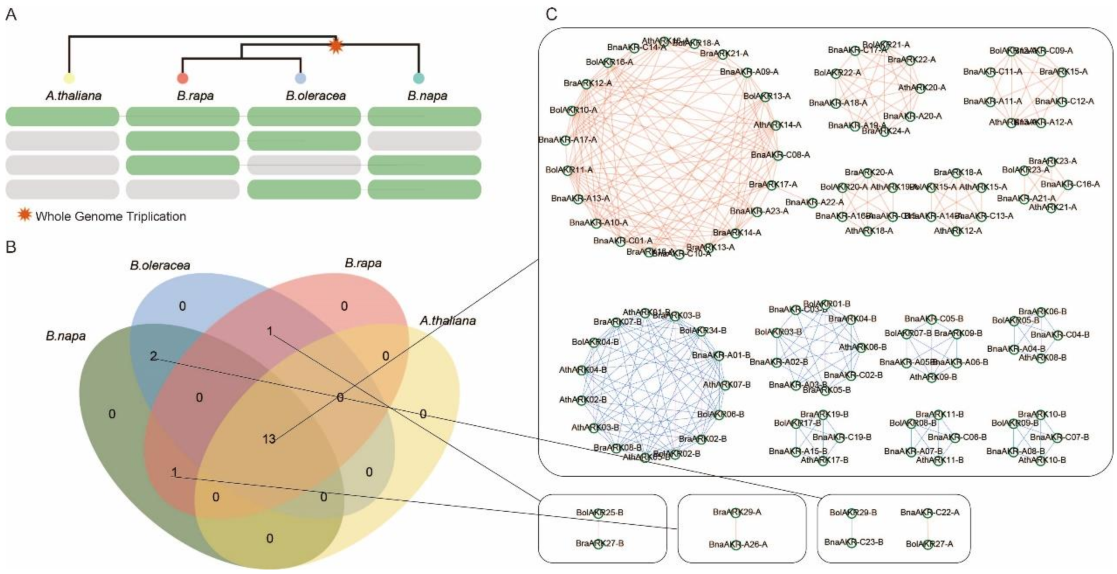

Figure S7. Relationship of aldo-keto reductase (*AKR*) homologous genes among *Arabidopsis thaliana, Brassica rapa, Brassica oleracea and Brassica napus*.

All of 17 cluster among AKR homologous genes were deduce by OrthoMCL program. (A) The distribution of AKR homologs clusters; (B) Venn diagram of AKR homologs relationship; (C) The interrelation network of AKR orthologs. The group A and B AKR orthologs gene pairs are highlighted by red and blue, respectively.
